# Smart Wearable Systems for Health Monitoring

**DOI:** 10.3390/s23052479

**Published:** 2023-02-23

**Authors:** Zhiyong Deng, Lihao Guo, Ximeng Chen, Weiwei Wu

**Affiliations:** 1 School of Nuclear Science and Technology, Lanzhou University, Lanzhou 730000, China; 2 Nuclear Power Institute of China, Huayang, Shuangliu District, Chengdu 610213, China; 3 School of Advanced Materials and Nanotechnology, Interdisciplinary Research Center of Smart Sensors, Xidian University, Xi’an 710126, China

**Keywords:** smart sensors, wearable systems, health monitoring, advanced materials

## Abstract

Smart wearable systems for health monitoring are highly desired in personal wisdom medicine and telemedicine. These systems make the detecting, monitoring, and recording of biosignals portable, long-term, and comfortable. The development and optimization of wearable health-monitoring systems have focused on advanced materials and system integration, and the number of high-performance wearable systems has been gradually increasing in recent years. However, there are still many challenges in these fields, such as balancing the trade-off between flexibility/stretchability, sensing performance, and the robustness of systems. For this reason, more evolution is required to promote the development of wearable health-monitoring systems. In this regard, this review summarizes some representative achievements and recent progress of wearable systems for health monitoring. Meanwhile, a strategy overview is presented about selecting materials, integrating systems, and monitoring biosignals. The next generation of wearable systems for accurate, portable, continuous, and long-term health monitoring will offer more opportunities for disease diagnosis and treatment.

## 1. Introduction

In modern medicine, diagnosis, monitoring, and therapy methods mostly rely on large-scale precise equipment, which is inconvenient and uncomfortable or even injurious to patients. Meanwhile, due to limited medical resources, the diagnosis and monitoring processes have gradually tended to become portable and domiciliary, which necessitates the development of miniaturized and portable equipment [1,2,3,4,5,6,7,8]. In recent years, numerous flexible and wearable electronic devices have been designed with the advent of the Internet of Things (IoTs), and these novel devices can be also used in the medical domain to constitute diagnosis/monitoring/therapy systems that possess many advantages, such as being non-invasive and lightweight, having skin conformability and portability [1,2,3,4,5,6,7], etc. Traditional devices and systems used to collect biosignals under normal life conditions are always strongly limited by large, repetitive deformations [8,9], such as joint movements (at elbows, knees, wrists, etc.) and skin stretching. In order to use the obtained signals as a basis for disease diagnosis and monitoring, the sensing performance of wearable diagnosis/monitoring devices, i.e., sensitivity, selectivity, robustness, instantaneity, etc., should be improved under deformation.

For wearable devices and systems to diagnose disease and monitor health, electrodes in wearable devices and systems need to have excellent conductivity and connect with an outstanding sensing platform during long-term use, while a stable skin–device conformable surface is also indispensable to maintain real-time and continuous monitoring [10,11]. Meanwhile, wearable devices and systems are pursuing thin thickness and small sizes for miniaturization and portability, which are necessary for improving conformability, but disadvantageous for their other properties such as conductivity, sensitivity, etc. For example, according to Joule’s law, the severe fever issue is a common problem in integrated circuits caused by smaller circuit sizes. Embracing this trade-off, then, is the kernel to constructing high-performance wearable disease diagnosis/health-monitoring devices [10]. With the assistance of advanced materials, flexible electronics, and nano-/microfabrication, there are many studies focused on sensing materials, flexible electrodes, or substrates in wearable devices and systems, and through integrating promoted units, the performance and size of wearable systems might be optimized simultaneously [12,13,14,15,16,17].

To date, many wearable devices and systems, e.g., smart watches, wristbands, headbands, eyeglasses, smart tattoos, and shoes, have been designed, fabricated, and commercialized [18,19,20,21,22,23]. In wearable systems, functional units can be categorized into a substrate, flexible electrodes, sensing materials, communication/energy units, and device-integrated modes between these units [3,24,25,26,27,28,29,30,31]. As mentioned above, in order to ensure the performance and comfort of wearable systems under deformation, design principles should include: (1) each unit and system possess excellent structure robustness, and their structure should not be easily destroyed under repetitive deformation, (2) the function of each unit and the connection between them should be stable and insusceptible to deformation, (3) the surface of wearable systems contacting with human skin should be conformable, nontoxic, harmless, and comfortable. Based on the first and second principles, many measures have been managed, which can be classified into two categories, i.e., materials and structures. As for materials, there are many novel conductive nanomaterials and polymers that possess inherent flexibility. These materials can maintain their performance under deformations and can be used as sensing materials or flexible electrodes [26,28,32]. The stability and robustness of wearable systems can also be achieved through adjusting the structure of devices and systems, known as stretchable structures. Structures such as wave, serpentine, helix, and kirigami have been demonstrated to provide effective strain release under deformations [23,33,34,35,36,37]. Moreover, integrated circuits, wireless transmission, and artificial intelligence can be introduced to further enrich the functionality of wearable systems to provide medical information, e.g., remote interaction and family practice [3,10]. Briefly, the collected biosignals are transferred to the analysis terminal via wireless transmission methods, such as Bluetooth, antenna, radio frequency identification devices (RFID), and near-field communications (NFC), which releases the monitoring systems from an unwieldy analysis terminal [38,39,40,41,42]. In addition, with the rise in wireless charging, energy can also be wirelessly transferred to wearable systems to achieve self-powered systems, and in addition to this, the energy supply process includes energy capture, conversion, and storage, including triboelectric nanogenerators (TENG), (organic) photovoltaic cells, ion batteries, and supercapacitors [43,44,45,46,47]. Machine learning, as a subset of artificial intelligence, has been used to analyse biosignals in many fields, and this high throughput method can simulate human brains in parallel information processing and enable wearable systems for signals analysis or even disease diagnosis.

**Figure 1:**
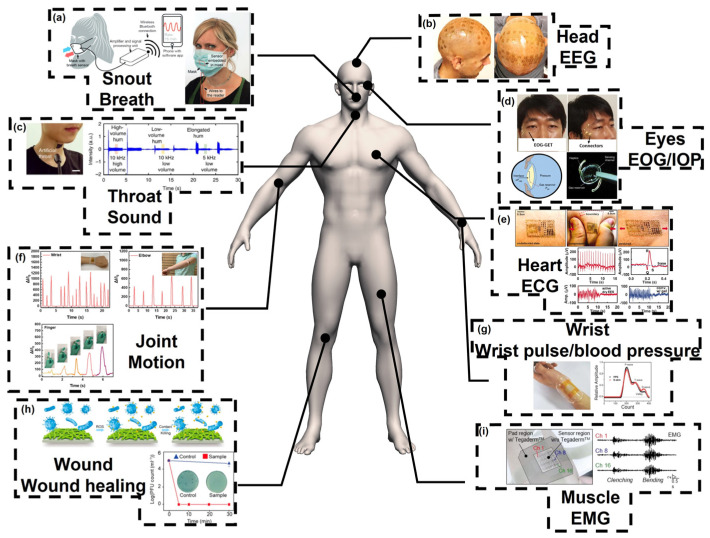
Wearable systems for human health monitoring, including (**a**) breath monitoring. Reproduced with permission from ref. [58]. Copyright 2016 Wiley-VCH. (**b**) Electroencephalogram monitoring. Reproduced with permission from ref. [59]. Copyright 2019 Nature Publishing Group. (**c**) Sound monitoring. Reproduced with permission from ref. [60]. Copyright 2017 Nature Publishing Group. (**d**) Electrooculogram and intraocular pressure monitoring. Reproduced with permission from ref. [61]. Copyright 2014 Nature Publishing Group. (**e**) Electrocardiograph monitoring. Reproduced with permission from ref. [23]. Copyright 2011 American Association for the Advancement of Science. (**f**) Movement monitoring. Reproduced with permission from ref. [62]. Copyright 2021 The Royal Society of Chemistry. (**g**) Wrist pulse and blood pressure monitoring. Reproduced with permission from ref. [63]. Copyright 2014 Wiley-VCH. (**h**) Wound healing monitoring. Reproduced with permission from ref. [64]. Copyright 2018 American Association for the Advancement of Science. (**i**) Electromyography monitoring. Reproduced with permission from ref. [65]. Copyright 2021 American Association for the Advancement of Science.

Nowadays, wearable systems are extensively used to monitor human basic physiological signals, such as oxygen saturation, wrist pulse, blood pressure, and heart rate. Researchers have demonstrated that wearable systems have great potential in disease diagnosis, therapy, drug delivery, etc., as shown in Figure 1 [26,29,48,49]. For instance, electrophysiology signals [23], i.e., electrocardiograph (ECG) [50], electroencephalograph (EEG) [51], electromyography (EMG) [52], and electrooculogram (EOG) [53], are collected via epidermal electronic devices, providing visual data for studies and treatment of cardiovascular and cerebrovascular diseases, and anatomical body movements can also be monitored by wearable systems. Moreover, the use of (bio)chemical biomarkers in body fluids and breath as diagnostic information to analyse the physiological function of the human body is a promising approach, and wearable (bio)chemical sensing systems have been designed, fabricated, and used to diagnose diseases such as diabetes mellitus, cancer, etc. Wearable diagnosis/monitoring/therapy systems can be utilized as a substitution for large-scale precise equipment during early diagnosis and monitoring of disease because of their painless, inexpensive, and portable advantages [54,55,56,57].

**Scheme 1:**
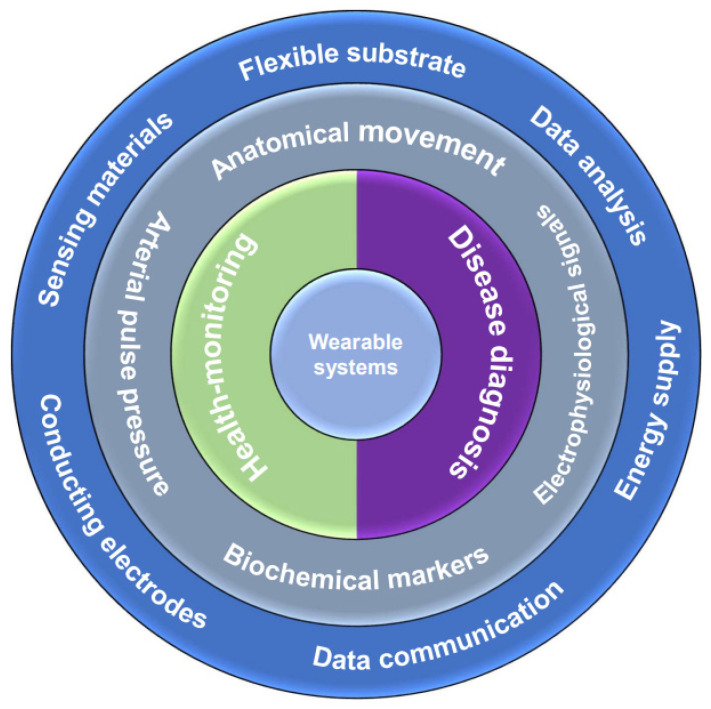
Schema of the main structure of this article. The aim (core) of this review is wearable systems for health monitoring and disease diagnosis (first ring). The practical applications of wearable systems in health monitoring are divided into four parts, i.e., anatomical movement monitoring, arterial pulse pressure monitoring, electrophysiological signals monitoring, and biochemical markers monitoring (second ring). The components of smart wearable health-monitoring systems are summarized, including flexible substrates, sensing materials, conducting electrodes, data communication systems, energy supply systems, and data analysis systems.

With the change in living environment, the incidence ages of chronic diseases, cardiovascular diseases, cervical spondylosis, etc., have tended to be younger, so daily monitoring and early diagnosis have gained increasing attention and demand. The designed and fabricated wearable diagnosis/monitoring systems have gradually become multi-functional, cross-disciplinary, and advanced. Thus, this review focuses on the wearable diagnosis and monitoring systems and combs the design strategy, including materials selection, structure design, and systems integration. We also highlight practical applications of wearable systems and conclude the challenges and future directions to develop the performance of wearable systems for disease diagnosis and health monitoring. In Scheme 1, we illustrate the research directions and current gaps, which also constitute the main structure and scope of our review. Firstly, the materials used in wearable health-monitoring systems are summarized, including flexible substrates, sensing materials, and conducting electrodes. Moreover, the integration of monitoring systems is discussed, focusing on data communication systems, energy supply systems, and data analysis systems. Secondly, wearable health-monitoring systems applied to anatomical movement monitoring, arterial pulse pressure monitoring, electrophysiological signals monitoring, and biochemical signals monitoring are concluded. Finally, concluding remarks and an outlook for further study are presented.

## 2. Materials and System Fabrication

Wearable diagnosis/monitoring devices and systems are complex and highly integrated. To fabricate promising wearable systems, materials selection and structure design are very critical [26,66]. Generally, the functional units of wearable systems include (1) stretchable substrate, (2) sensing materials, (3) flexible electrodes, and (4) systems integration [29]. Moreover, the performance of systems is determined by the function and interactions of each individual unit, so we must consider individual units as well as the entire system.

### 2.1. Flexible Substrates

Traditional rigid substrate materials, such as SiO_2_, Si, and ITO/FTO, only show mechanical flexibility when the thickness of these materials reduces to dozens of nanometres. However, the mechanical strength is also weakened simultaneously [67]. Therefore, stretchable substrates that possess excellent mechanical flexibility and strength are demanded to maintain the stability of systems under complicated deformation [68,69]. Many elastic polymers, e.g., polyethylene terephthalate (PET) [70], polydimethylsiloxane (PDMS) [71], polyethylene naphthalate (PEN) [72], polyimide (PI) [73], poly(styrene-ethylene-butylene-styrene) (SEBS) [74], etc., have been widely chosen as flexible substrates for wearable systems due to their intrinsic mechanical flexibility. Moreover, most elastic polymers are easily processable and nontoxic, which reduces the risks in biomedical use [75].

The choice in substrate mainly depends on the target physiological signals and the placement of flexible devices. When monitoring different target physiological signals, flexible substrates need to possess different properties. For example, flexibility and stretchability are the most crucial properties of flexible substrates used in movement-monitoring systems, but permeability is the chief property which needs to be considered when wearable systems are used to monitor biochemical markers contained in breath or body fluids. Moreover, non-stretchable substrates are preferable in some monitoring systems (such as biomarker-monitoring systems) since stretchable substrates may induce the mechanical destruction of sensing layers. In addition, wearable systems always suffer serious deformation, which may cause them to break down when they are placed near joints (e.g., elbows, wrists, knees), so some special properties should be endowed to flexible substrates, such as self-healing. Accordingly, the common flexible substrates are categorized here based on their characteristics.

PI and PET are two commercial polymers with high tensile strength, excellent bendability, low cost, and great chemical stability. Since PI can withstand high temperature (up to 452 °C), weak acid, alkalis, and organic solvents, this material is widely used in Micro-Electro-Mechanical Systems (MEMS). A mass of flexible devices based on PI substrates have also been fabricated for pressure and bend sensing [76,77]. However, the yellow colour of PI hinders its use in many applications, including solar cell and flexible display, where high light transparency is required [73]. PET, which also offers satisfactory physical and chemical stability, is a transparent flexible substrate that allows more than 85% of light to pass through. This material can be used as a substrate for solar cells or flexible displays to replace PI [72,78]. In general, PET and PI can only be bent but not stretched. There are two strategies for fabricating stretchable substrates: constructing stretchable topological shape structures (described in the subsequent section) and using stretchable materials.

The stretchability of PVA and PDMS makes them popular as stretchable substrates in wearable systems, which can be stretched over a 1000-fold stretch [79]. Moreover, due to the liquid state before solidification, PDMS and PVA can be easily processed into various shapes with different moulds, e.g., hemispheric, pyramid, ripple, etc. [66,80,81,82], and these moulds are not only delicate moulds produced by lithography but also common microstructured items such as abrasive paper and lotus leaves [63,83]. Microstructured substrates can effectively improve the sensing performance of wearable systems [66,84,85,86]. For example, Cheng et al. imitated the microspine receptors in human skin and spin-coated MXene on microstructured PDMS, which was constructed using abrasive paper as a mould, and the sensitivity and detected limitation of the obtained pressure sensor were related to the roughness of the abrasive paper, so the optimized flexible pressure sensor could achieve high sensitivity (151.4 kPa^−1^) and low detected limitation (4.4 Pa) [83]. The morphology, size, and density of microstructures on a substrate all influence the performance of wearable systems. These factors should be precisely controlled using MEMS technology, e.g., lithography and reactive ion beam etching. More interestingly, the special microstructured PDMS substrate can detect pressure direction, which is significant but difficult because the pressure always coexists with shear forces [87]. This method will be discussed in the subsequent section.

Furthermore, PVA has a self-healing property due to abundant hydrogen bonds inside, which makes wearable systems with PVA substrate resistant to damage [88]. Mechanical failure is inevitable and fatal during the use of wearable systems, but like human skin, substrates using self-healing materials can self-heal when they are mechanically broken [89,90]. The self-healing mechanisms of these materials include hydrogen bonds, chelation, and electrostatic interaction. Advanced self-healing materials have been proposed to lengthen the lifespan of wearable systems. For instance, Huynh et al. provided a flexible self-healing device based on polyurethane (PU) and gold nanoparticles for pressure, temperature, and gas sensing, and the performance of the device can be maintained even after 4–5 scratch/healing cycles [89,91]. A PVA-functionalized single-walled CNT-polydopamine sensor was explored by Wan and coworkers, which has high self-healing efficiency (99%) and fast ability (<2 s), and this sensor has an excellent performance in healthcare monitoring [92].

Fibres and textiles possess excellent breathability that the abovementioned substrate materials lack, so they are supposed to be the natural materials closest to human skin. Silk has been widely reported as a substrate in flexible and wearable electronic products owing to its many benefits [93,94], including biocompatibility, transmissivity, mechanical robustness, etc. As common fibres and textiles are insulating, they need to be functionalized when used as a substrate in wearable systems [95,96]. Two methods are mainly used to functionalize the textiles: integrating external devices (sensors, power sources, conductive pathways/electrodes, etc.) on textiles and spinning conductive or sensing fibres in textiles [97,98,99]. Both methods have their advantages; there are various smart wearable substrates developed on textile substrates, and functionalized textiles bring traditional cloths to the smart, high-tech, and multi-functional stages [100].

### 2.2. Sensing Materials and Conducting Electrodes

#### 2.2.1. Metals and Traditional Inorganic Materials

Sensing materials and conducting electrodes are two of the most crucial components in wearable systems. The selective principle of electrode materials mainly focuses on their conductivity and robustness, but the sensing materials should be chosen according to the aim function [26,28,101]. The conductivity and function of both the two units have to be stable under repeated deformation to ensure the stable performance of systems, and to avoid the interference of sensing signals, the electrode resistance should be insensitive to deformation.

Metals, e.g., Au, Ag, Cu, Pt, and some traditional inorganic materials, e.g., indium tin oxide (ITO) and fluorine doped tin oxide (FTO), have been utilized as conducting electrodes because of their excellent conductivity [102,103]. In terms of sensing materials, the materials vary depending on their purpose of use, such as transition metal oxide (semiconductor) for optical sensing [104], Pt and Mg (metal) for temperature sensing, and Au and Ag for force sensing [105,106]. These metals and traditional inorganic materials have been widely used in rigid devices but due to the inherent rigidity of these materials, they are easily broken under deformation. Even though some metals are soft, there is still permanent cumulative damage, i.e., cracks and breakage, under cyclic stress or strain [107,108,109]. In wearable systems, rigid devices and electrodes are attached to or buried in flexible substrates, but via theoretical simulation and experimental test, the modulus disparity of devices and flexible substrate causes stress concentration, which aggravates the deformation and damage of rigid devices [52,110,111,112]. Metals and rigid inorganic materials can be made more flexible in two ways: by reducing the thickness of the materials or by constructing stretchable structures [23,38,113]. The strain energy per area can be effectively brought down by reducing the thickness of materials. This strategy makes materials more flexible, and the special stretchable structures, such as serpentine, sigmate, and wave, can disperse stress distribution, thereby avoiding stress concentration caused by deformation [38,67].

**Figure 2:**
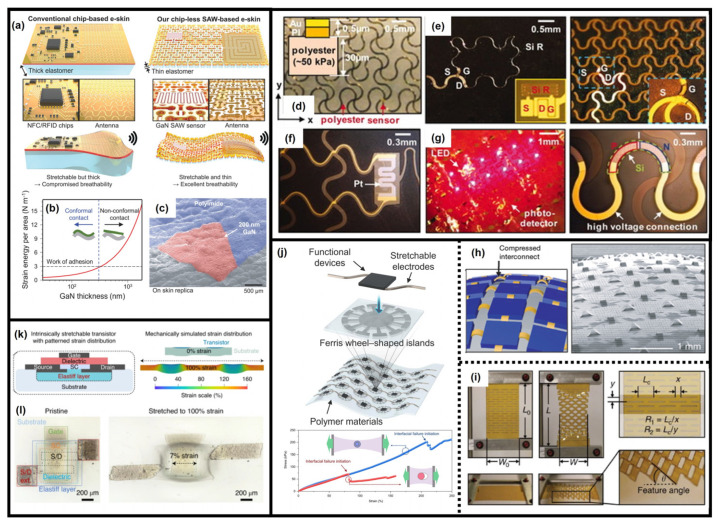
The typical design strategies of conducting metals and traditional inorganic materials in wearable systems. (**a**) Schematic of the wearable system with rigid devices and chip-less wearable system. (**b**) Estimation of minimum sensing material thickness required for conformal lamination on human skin. (**c**) SEM image of conformal contact between material and skin. Reproduced with permission from ref. [38]. Copyright 2021 American Association for the Advancement of Science. (**d**) Optical micrograph of an epidermal electronic skin (EES) with a filamentary serpentine (FS) design. (**e**) An active electrophysiological sensor part, (**f**) temperature sensor part, and (**g**) LEDs and photodetectors part of an FS-EES. Reproduced with permission from ref. [23]. Copyright 2021 American Association for the Advancement of Science. (**h**) Illustration and SEM image of an island–bridge fabrication strategy. Reproduced with permission from ref. [114]. Copyright 2008 Nature Publishing Group. (**i**) Response of kirigami structure to stretching in the axial direction. Reproduced with permission from ref. [115]. Copyright 2015 Nature Publishing Group. (**j**) Schematic illustration of stretchable electronics with the Ferris wheel-shaped island (FWI) array in Ecoflex and stress versus strain for the circle-shaped island (red trace) and FWI (blue trace) in Ecoflex under stretching. Reproduced with permission from ref. [116]. Copyright 2022 American Association for the Advancement of Science. (**k**) Two-dimensional diagram showing a representative transistor structure. (**l**) Optical microscope images of one transistor under 0% (left) and 100% (right global strain). (**k**,**l**) Reproduced with permission from ref. [117]. Copyright 2021 Nature Publishing Group.

Recently, Kim et al. designed a GaN surface acoustic wave device (Figure 2a) [38], and they proposed that the contact mode between thick GaN and substrate is non-conformal contact because of high strain energy per area, whereas when GaN is thinner than 200 nm, the strain energy can be reduced to less than 3 N/m (work of adhesion) and the contact converts to conformal contact (Figure 2b). As shown in Figure 2c, the surface acoustic wave of a 200 nm GaN sheet which conformally contacts with PI substrate and skin replica is exceedingly sensitive to skin deformation, and this GaN is also sensitive to ultraviolet light and Na^+^ ion because of its inherent semiconducting property. This GaN sensor is connected to an antenna that is wireless for signals and energy transmission, and the conducting pathway and antenna are composed of serpentine Au, which represents another method mentioned above.

Rogers et al. provided an epidermal electronics skin (EES) [23], and the interconnect line in this system was formed into a large-amplitude serpentine shape, shown in Figure 2b, referring to the open-mesh structures [118,119]. The effective modulus (*E*_EES_) and bending stiffness were proved to be paramount, rather than the range in stretchability, and the effective modulus was approximately expressed as *E*_EES_ = *E*_int_ (1 + *L*_d_/*L*_s_), where *E*_int_ corresponds to the effective modulus of the interconnect line, *L*_d_, and *L*_s_ is the size of devices and distance between devices, respectively. Introducing a filamentary serpentine interconnect line reduced the effective value of *L*_d_, and with thinner devices, the effective modulus of EES was minimized, which also reduced the driving forces of this EES for interface delamination and avoided device failure under deformation. Through finite element modelling, the stress on interconnect was evenly scattered over the sinuosity of the serpentine interconnect line, minimizing the stress concentration, which may cause mechanical breakage. Based on these serpentine interconnect lines, multiple devices were fabricated, such as an electrophysiological sensor (Figure 2e), a temperature sensor (Figure 2f), a photo-detector, and AlInGaP LEDs (Figure 2g). By integrating these sensors and devices, a flexible multi-functional system was obtained, which can be used for long-term electrophysiological (ECG, EEG) monitoring. A similar island–bridge interconnect strategy is also used in non-plane devices [120], such as a hemispherical electronic eye camera demonstrated by Rogers et al. Rigid silicon devices (p–n diode photodetector) islands were interconnected by compressed Au electrodes (Figure 2h) [114]. The stress caused by deformations was induced to distribute on flexible interconnect lines, easing the strain on rigid devices and reducing the risk of damage.

As mentioned above, some flexible substrates cannot be stretched, which limits their applications. Inspired by Chinese traditional paper art, i.e., origami and kirigami, which transform two-dimensional (2D) paper into a three-dimensional (3D) structure, designing the substrates in special patterns can endow them with stretchable property [30]. When the axial strain is applied to non-stretchable substrates such as PI, PET, and paper, the stress is concentrated inside the substrate, but by transforming these substates into kirigami structures, stress and stretching energy can be dissipated by opening the kirigami pattern, thus allowing the flat substrate to bend out of the plane (transform 2D into 3D) [35,37,113,121]. As shown in Figure 2i, kirigami patterns of substrates can be elaborately designed via finite element modelling, and the substrate pattern parameters, e.g., incision length *L*_c_, incision horizontal separation distance *x*, and vertical distance *y*, determine the length, width, and feature angle of opening substrates; in another word, stretchability [115]. By optimizing the kirigami structure, the substrate and electronics can achieve a tensile strain of 215% [37].

Embedding rigid devices into soft polymer substrates (e.g., Ecoflex, PDMS, and Ecoflex Gel) is a strategy to minimize the lateral strain on the devices, because soft polymers with low elastic modulus are generally stretchable under deformation [34,74,122]. However, the mismatched elastic modulus of soft polymer substrates and embedded rigid devices is a double-edged sword that can both protect rigid devices and lead to crack propagation along the interface between the substrate and the devices [111,112]. More recently, Yang et al. presented geometrically engineered rigid islands which were fabricated into Ferris wheel patterns to suppress cracks at the interface between soft polymer substrates and embedded rigid islands (Figure 2j) [116]. Theoretical simulations and experimental observations showed that the interlocking Ferris wheel structure of the rigid devices anchored the soft polymer and improved failure strain under stretching, thus extending the fatigue life of wearable systems under practical usage. Moreover, another way to integrate devices and substrates is to attach the devices above the substrate [11,123,124]. However, when the system is stretched, the applied strain causes the stress to concentrate on the devices because the rigid devices and soft polymer substrates have different elastic moduli. Consequently, the performance of such wearable systems is invariably compromised by mechanical strain during use, limiting their practical applications, such as in physiological signal monitoring and analysis. This limitation was addressed by Bao and coworkers with a strain-insensitive, stretchable transistor array by introducing a stiff layer (named elastiff layer) between rigid devices and soft substrates (Figure 2k) [117]. The elasticity of the introduced stiff layer was controlled by varying the crosslinking density of elastomer substrates in special areas, and the stress induced by mechanical strain concentrated on the elastiff layer under stretching, but due to the stiffness of the elastiff layer, the stress could not be delivered to the above devices [125,126,127,128,129]. The mechanical strain distribution on the substrate and devices was simulated by finite element modelling. The simulation showed that under the protection of the stiff layer, the strain on the active device area was reduced to less than 5%, even when the system was stretched up to 100%. To confirm the theoretical result, stretchable device arrays were fabricated as shown in Figure 2l. As a result, when stretched to 100% strain, the local strain was 7%, making this strain-insensitive stretchable system suitable for monitoring electrophysiological signals.

#### 2.2.2. Conductive Polymers

Compared with metals and traditional inorganic materials, polymers possess inherent flexibility/stretchability, biocompatibility, and stability. However, polymers are usually not used as electrodes because of their poor conductivity [32]. All-polymer devices have attracted much attention in long-term health-monitoring and wearable systems, and improving the conductivity of the polymer used in functional units is key. Conductive polymers can be divided into two types: intrinsically conductive polymers and conductive polymer composites (described in the subsequent section) [130,131]. However, wearable systems based on intrinsically conductive polymers, such as poly(ethylenedioxythiophene):poly(styrenesulfonate) (PEDOT:PSS), perform poorly and are unstable because many intrinsically conductive polymers are insufficiently stretchable/flexible and adherent [132].

**Figure 3:**
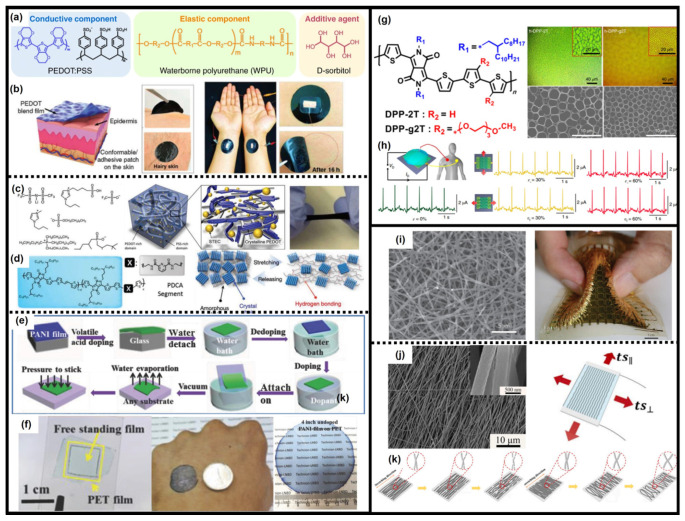
The conductive polymer and nanowire networks used in wearable systems. (**a**) Chemical structures of PEDOT:PSS, WPU, and D-sorbitol. (**b**) Schematic illustration and optical photo of a blend film dry electrode. Reproduced with permission from ref. [132]. Copyright 2020 Nature Publishing Group. (**c**) Chemical structure of PEDOT:PSS and schematic diagram of PEDOT:PSS film with stretchability and electrical conductivity enhancers. Reproduced with permission from ref. [134]. Copyright 2017 American Association for the Advancement of Science. (**d**) Chemical structure and schematic illustration of polymer under stretching and release state. Reproduced with permission from ref. [135]. Copyright 2016 Nature Publishing Group. (**e**) Flow chart of the self-supporting PANI film fabrication process. (**f**) Photograph of a freestanding PANI film. Reproduced with permission from ref. [136]. Copyright 2017 Wiley-VCH. (**g**) Molecular structures of DPP-2T and DPP-g2T polymers and optical (**top**) and SEM (**bottom**) images of honeycomb DPP-2T and DPP-g2T films. (**h**) Illustration of ECG recording setup and spontaneous heart activity measured on a volunteer with honeycomb films under different strains. Reproduced with permission from ref. [137]. Copyright 2022 Nature Publishing Group. (**i**) SEM image of randomly stacked electrospun nanofibres and photograph of an integrated sensor array attached to the surface of a soft balloon. Reproduced with permission from ref. [138]. Copyright 2016 Nature Publishing Group. (**j**) SEM image of ATO nanofibres and directions of the tensile strains applied to the ATO strain sensor. (**k**) The unidirectional sensing mechanism of the ATO strain sensor. Reproduced with permission from ref. [139]. Copyright 2022 Elsevier B.V.

Greco et al. introduced ethyl cellulose into PEDOT:PSS to obtain an adhesive ethyl cellulose/PEDOT:PSS bilayer EMG monitoring system, but this bilayer sensor is still non-stretchable, which results in the EMG signals being susceptible to mechanical strain caused by muscle movement [133]. For long-term health monitoring, Zhang et al. developed a full-polymer dry electrode by adding waterborne polyurethane (WPU) and D-sorbitol to PEDOT:PSS to create a self-adhesive (Figure 3a), biocompatible, and stretchable blend [132]. The conductivity of the obtained blend film depends on PEDOT:PSS, which builds conductive networks in the elastic component WPU, so the dry film electrode is stretchable and conductive in its entirety. This film can achieve firm adhesion to human skin (adhesive force > 0.41 N/cm), and a stable monitoring system for 1 month of continuous ECG signal monitoring can be assigned (Figure 3b). The same strategy was used by Wang et al. (Figure 3c), and the polymer films based on PEDOT:PSS exhibit high conductivity of 3100 S/cm (over 4100 S/cm under 100% strain), which can be maintained at 3600 S/cm after 1000 cycles to 100% strain [134].

Furthermore, attempts are made to modulate the conductivity of stretchable polymers for diverse usage requirements [140,141,142]. The fully flexible polymer thin-film field-effect transistor is frequently used in stretchable electronic devices for wearable systems, and the semiconductor is an element in the field effect transistor (FET) which relies on an intrinsic semiconducting stretchable polymer [143]. Oh et al. proposed that the stretchability of polymers can be enhanced by infusing more flexible molecular building blocks into conjugated polymers containing modified side-chains and segmented backbones, and thus they developed an intrinsically stretchable semiconducting polymer transistor [135]. As shown in Figure 3d, stretchability is conferred with intrinsically semiconducting polymers by designing a dynamic non-covalent crosslink moiety that dissipates energy through breaking bonds under strain. The FET based on this stretchable polymer maintains stably high field-effect mobility performance even after 100 cycles to 100% strain. Hence, regulating the chain component and structure of the polymer is a feasible way to improve the stretchability of intrinsically conductive polymers and to introduce an elastic component to them.

An ultra-thin polymer film is also flexible like inorganic thin-film materials, so by reducing the thickness of the polymer they can become more flexible. However, the ultra-thin polymer film deposited or spin-coated on a rigid substrate is difficult to be peeled off, and, thus, the choice of substrate is limited [144,145,146]. Wu et al. reported a scalable fabrication approach to synthesize freestanding, transparent, and ultra-thin (190 nm) flexible polymer films [136]. The synthesis process is shown in Figure 3e, and an ingenious peeling method was designed for the transfer of the polyaniline (PANI) polymer to any substrates, including PET, PE, and even human skin. Moreover, the conductivity and band gap of the obtained PANI film can be easily tuned by doping, and due to the ultra-thin thickness, the conductivity of the flexible film is uninfluenced by a mechanical strain. As a demonstration, a volatile organic compounds (VOCs) sensor was built using this ultra-thin PANI film, and this flexible sensing system could detect and distinguish various VOCs with different acid-doped PANI films even under bending/strain states.

Recently, Chen et al. reported a highly stretchable organic electrochemical transistor (OECT) device, in which a honeycomb morphology film is combined with a semiconducting polymer. The poly(2,5-bis(2-octyldodecyl)-3,6-di(thiophen-2-yl)-2,5-diketo-pyrrolopyrrole-alt-2,5-bis(3-triethyleneglycoloxy-thiophen-2-yl) (DPP-g2T) can self-form a honeycomb structure during the solvent evaporation process because of its amphiphilic property (Figure 3g), and this honeycomb structure film possesses excellent stretchability with negligible performance degradation, even under stress strain up to 140% [137]. Finite element analysis revealed that in the honeycomb structure, the strain distribution is dispersed and weakened (maximum principal strain <60% under 89% strain) compared with uniform stain distribution in dense films, and this phenomenon is similar to sponge behaviour in real life. Due to its stable structure, the electrical pathway and response of this architecture are suitable for the amplification and monitoring of electrophysiology signals (Figure 3h) [147]. In addition, the breathability of this porous architecture is obviously superior to that of dense films, which improves wearing comfort and reduces rejection. The strategy can also be adapted to the design of wearable body fluid analysis systems. Many other impactful approaches to enhance the flexibility/stretchability of conductive polymers have also been put forward, such as using an out-of-plane strategy and a kirigami structure. Margaritondo et al. constructed 3D PEDOT:PSS nanoarches with 270% stretchability [148]. Similar porous sponge structures can also be obtained via a sugar template, and Lo et al. used PDMS as a flexible substrate and constructed a PEDOT:PSS conductive network [149]. Due to the ample contact with the gel, this wearable device is accurate in recording patients’ electrophysiology signals.

#### 2.2.3. 1D Nanowire/Fibre/Tube Networks

Compared with bulk materials, 1D nanowires/fibres/tubes have excellent flexibility/stretchability [150,151]. The 1D materials can disperse stress and/or slip under strain to reduce mechanical damage, which makes them a suitable candidate for wearable systems [152]. Numerous 1D material networks have been applied, including metal (e.g., Au, Ag, Cu) nanowires, carbon nanofibres/tubes (CNF/CNT), electrospun polymer fibres, and other 1D materials which satisfy multifarious application needs [49,153]. The 1D material network is similar to natural fabrics and has great application potential in wearable devices/systems [154]. Moreover, 1D materials can stack and form a porous network that is lightweight, air permeable, and flexible, allowing wearable devices/systems based on 1D nano-wires/fibres/tubes to possess better comfort and mechanical robustness [155].

For instance, as shown in Figure 3i, a pressure sensor was fabricated by Lee and coworkers using CNT. This sensor is flexible, transparent, and bending-hyposensitive [138]. The low bending sensitivity of this sensor is due to the nanoporous structure built by 1D CNTs because they slip under bending-induced strain, while under pressure, the contact area of each nanotube changes, maintaining high pressure sensitivity. A wearable bending-insensitive pressure sensing device was designed using 1D CNT network film to accurately measure the distribution of pressure (Figure 3i right), and the properties of the sensor remained unchanged even though the sensor was bent to a large extent (bending radius <80 μm). This simple strategy inspired many other researchers concerning wearable systems based on 1D nanowire/fibre/tube devices and their health monitoring applications. A similar piezoresistive sensor was fabricated using carbon-decorated fabric fibre as the sensing material. Once the conductive fibre was pressed under pressure, the conductive pathway changed [156]. The fantastic performance of this sensor is comparable to that in clinical settings and commercial devices in health monitoring.

Electrospinning is a handy way to process polymers into nanofibre networks, and many novel electrospinning methods have been investigated to make polymer nanofibre networks with different kinds of structures [157]. The good insulation of many polymers makes them suitable as dielectric materials. Lin et al. reported a tactile sensor based on core–shell PDMS ion gel/ PVDF-HFP nanofibre mats, and this sensor responded to static and dynamic pressure via a piezocapacitive mechanism [158]. The thickness of nanofibre mats changed their capacitance under mechanical deformation, making this sensor capable of measuring pressure. Furthermore, the core–shell PDMS ion gel/PVDF-HFP nanofibre sensor was also self-powered. Finally, a demonstration of using the wearable pulse rate detector as a heart rate indicator was carried out.

In addition to piezoresistive and piezocapacitive sensing, the breakage process of nanofibres can also be used to sense strain. For instance, Yang et al. recently proposed an antimony-doped tin oxide (ATO)-oriented nanofibre film strain sensor [139]. The prepared ATO nanofibre is flexible and highly conductive, and the oriented nanofibrous structure can distinguish strain direction, i.e., the strain sensor exhibits high a gauge factor (GF) (up to 250) along nanofibre orientation direction with negligible GF (1.2) in the transverse direction. Briefly, when a mechanical strain was applied along the nanofibre orientation direction, the nanofibres easily broke, inducing evident resistance change, while in the transverse direction, the flexible nanofibres could slip under strain and the conductivity was maintained [159,160,161]. Moreover, this strain sensor was used as a wearable electronic for monitoring sophisticated human body motions.

#### 2.2.4. Composite Materials

Depending on the purpose of use, the different units of wearable devices/systems are generally composed of different materials, but the divergent physical properties of these materials (e.g., elasticity modulus, stiffness, and stretchability) can lead to severe problems that may interfere with their proper functioning [34,74,122]. As recommended above, for example, the mismatched elasticity modulus of sensing materials or electrodes and flexible substrates always results in an abominable interface separation. This invalidates the function of wearable systems [117]. Adding an adhesive layer can minimize the mismatch between the active layer and the flexible substrate. However, material selection for an adhesive layer should reconcile the properties of both two layers, which is challenging. Therefore, composite materials are put forward to ameliorate the matter [162]. Compared with intrinsically conductive polymers, composites applied in wearable systems are constituted by flexible/stretchable polymer matrices (e.g., PDMS, PVA, paper, hydrogel) and conductive material additives (e.g., metal nanoparticles, liquid metal, 1D/2D materials), which are equipped with better conductivity and flexibility/stretchability [163].

##### Metal Composites

**Figure 4:**
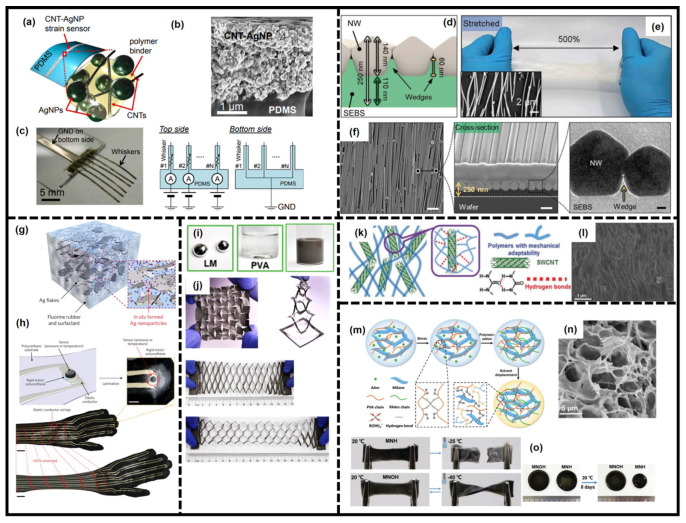
Design strategies of composite materials used in wearable systems. (**a**) Schematic of a CNT-Ag nanoparticle composite film patterned on a PDMS substrate. (**b**) Cross-sectional SEM image of the CNT-Ag composite film. (**c**) Optical image of a fully fabricated e-whisker array. (**d**) Circuit diagrams of the e-whiskers. Reproduced with permission from ref. [164]. Copyright 2013 Proceedings of the National Academy of Sciences of the United States of America. (**d**) Schematic illustration of the stretchable composite membrane cross-section. (**e**) Photographs of a free-standing membrane under 500% strain. (**f**) SEM and TEM images of closely packed nanowires in the membrane. Reproduced with permission from ref. [65]. Copyright 2021 American Association for the Advancement of Science. (**g**) Schematic illustration of the Ag polymer composites with in situ-synthesized Ag nanoparticles. (**h**) Structure and photograph of wearable system based printable Ag polymer composite material. Reproduced with permission from ref. [131]. Copyright 2017 Nature Publishing Group. (**i**) Photograph of composite material consisted with liquid metal and PVA. (**j**) Different kirigami patterns of composite material paper. Reproduced with permission from ref. [165]. Copyright 2022 American Chemical Society. (**k**) Schematic illustration of soft thermal responsive materials. (**l**) SEM image of thermal responsive materials with 20 wt% of CNT to polymers. Reproduced with permission from ref. [166]. Copyright 2016 Wiley-VCH. (**m**) Schematic illustration of the fabrication of a MXene nanocomposite hydrogel (MNOH). (**n**) SEM image of the freeze-dried MNH. (**o**) Photographs of the anti-freezing (**left**) and long-lasting moisture retention (**right**) performance of MNOH. Reproduced with permission from ref. [88]. Copyright 2019 Wiley-VCH.

Takei et al. reported a highly sensitive composite film electronic whisker [164]. The composite film is based on CNTs, silver nanoparticles, and polymers, as illustrated by Figure 4a. The conductive network matrix formed by nanotubes and nanoparticles has excellent conductivity, while the polymer binder ascertains the stability of the network matrix under deformations (Figure 4b). The resistivity and sensitivity of these composite films are controllable through modulating the ratio of components, which can achieve ultra-high sensitivity up to 8%/Pa, and the composite film was fabricated into a whisker form, further aggravating the strain and enhancing its sensitivity (Figure 4c). The elastic matrix based on polymer binders can not only bond the sensing inorganic components, but also stabilize the adhesion between the sensing layer and substrate, improving the flexibility/stretchability of systems. This flexible ultra-highly sensitive pressure sensor may possess wide potential applications in human–machine interfacing and wearable health-monitoring systems.

It is common for conductive materials in an elastic matrix to be disordered, and the properties (e.g., conductivity) of the composite to be isotropic. More recently, Jung et al. proposed a new idea for fabricating a conducive and elastic nanomembrane with aligned Ag nanowires using a floating assembly method [65]. The originally disordered Ag nanowires were dragged by Marangoni flow induced by a surface tension gradient (Figure 4d). In brief, water-immiscible solvents (i.e., toluene) and water-insoluble elastomers were spread on water/ethanol mixtures containing amphiphilic ligands [e.g., polyvinyl pyrrolidone (PVP)], which reduced the interfacial energy between toluene–elastomer and water–ethanol. With the dissolution of ethanol, the local surface tension gradually decreased, resulting in a surface tension gradient and Marangoni flow. This float assembly phenomenon occurs commonly in nature, which can be applied to fabricate various elastic nanocomposite membranes, and the conductive materials include Ag nanowires, Au-Ag core–shell nanowires, Ag nanoparticles, and Au nanoparticles, while the elastomers can be SEBS, thermoplastic polyurethane (TPU), and poly(styrene-isoprene-styrene) (SIS). As a consequence, this approach effectively distributes the strain on the membrane, which improves its elasticity under high loading conditions (Figure 4e), and the cold-welding stage thereafter ensures excellent conductivity (Figure 4f). In addition, a directional strain sensor can be designed based on this elastic membrane with aligned nanowires. As mentioned above [139], resistance changes under deformation in parallel and vertical directions differ by order of magnitude. This anisotropy of conductivity can also be eliminated through perpendicularly stacking two membranes. Based on this highly elastic, conductive, and multifunctional composite membrane, a multifunctional epidermal sensor can be fabricated, and this wearable system can measure temperature, humidity, strain, and electrophysiological signals simultaneously.

Matsuhisa et al. used Ag flakes and in situ formed Ag nanoparticles as conductive additives, and fluorine rubber and methylisobutylketone (MIBK) as elastic polymer matrices in constituting printable elastic conductors (Figure 4g) [131]. The Ag flakes dispersed in fluorinated elastomer had excellent conductivity even under high strain, while the introduced Ag nanoparticles possessed a sensitive response to the surroundings (e.g., mechanical strain), enabling the initial conductivity of this composite to reach 6168 S/cm, which remains 935 S/cm at 400% strain [52,167,168]. The printable fluorinated elastomer enables the composite to be processed into various patterns to meet the needs of applications, and a fully printed sensor was demonstrated for pressure and temperature sensing (Figure 4h) [169]. Furthermore, the stretchable sensor can keep accurate sensing even when the applied strain is up to 250%. As a result, the favourable flexibility and stretchability of composite make the material a suitable basis for manufacturing wearable systems that can monitor human motions even in flexible areas such as elbows and knees.

Liquid metal (LM) [e.g., Ga-In eutectic alloy (EGaIn)] can serve as a conductive material in wearable devices/systems. Since LM behaves like a liquid, it rarely suffers from a broken issue under deformation [170]. However, precisely because of its flow behaviour, the usage of liquid metal is inconvenient, and requires extra equipment or methods, such as microfluidic channels and supplied bottom fibre [171]. Li et al. reported a way to prepare LM paper. In brief, the LM was ultrasonically dispersed into a CNF solution and blended with PVA, and then the composites were cast on paper (Figure 4i) [165]. The established LM conductive pathway was stabilized by the CNF/PVA matrix, and via kirigami-structure design (as mentioned above), the LM paper became stretchable, self-supporting, stable, conductor-exposing, and recyclable (Figure 4j). Moreover, the high conductivity endowed by LM enables this LM paper to be fabricated to monitor high-quality electrophysiological signals, (i.e., ECG, EEG, and EMG), and it can also be used as a self-powered wearable sensor after integrating with TENG.

##### Non-Metal Composite Materials

The combination of different materials increases the functionality of composite materials. Inspired by natural organisms, materials with excellent flexibility and self-healing ability are widely used as the elastic matrix to endow wearable systems with self-healing capacities [90,172,173]. However, elastic polymer materials rarely possess great conductivity and self-healing properties at the same time. Adding the conductive additives to form a conductive network in the elastic matrix can improve conductivity and maintain self-healing properties simultaneously [89]. Yang et al. combined CNT with mechanically adaptable polymers, and the CNT was functionalized with carboxyl groups which can interact with hydrogen bonds in the polymer matrix (Figure 4k) [166]. Briefly, the adaptable polymers can self-heal after cutting and the internal conductive network can be also repaired due to the strong interaction between additive and matrix (Figure 4l). In addition, the composite material consisting of conductive CNT and elastic polymer matrix possesses good electrical conductivity, and a soft thermal sensor can be fabricated based on this composite. The resistance of this thermal sensor decreases with increasing temperature because the segments of polymers which have low glass-transition temperature disentangle from CNT upon heating and the intercontact of CNT becomes better, reducing the resistance of the thermal sensor [174]. This flexible self-healing thermal sensor effectively improves the service life of the sensor and has great potential in artificial intelligence robots or wearable systems.

Many flexible and wearable devices have been designed using conductive hydrogels in recent years due to their excellent conductivity, mechanical properties, and biological characteristics [175,176,177]. Because of the abundant hydrogen bonds, many hydrogels are mechanically adaptable, such as polyacrylamide (PAAM) and polyvinyl alcohol (PVA). However, the inevitable loss of water is a huge obstacle for their applications, and with water evaporation, the function of the structure of devices/systems based on hydrogels suffers from catastrophic collapse [178,179]. Liao et al. provided a facile solvent displacement method to prepare a flexible, anti-freezing, conductive wearable hydrogel sensor [88]. The hydrogel polymer matrix consists of PVA and PAAm, and the conductive additive is MXene nanosheets (Figure 4m,n). Composite materials were soaked in ethylene glycol (EG) solution to partially replace the water in hydrogel polymer networks, enhancing moisture retention (8 days), and thereby resolving water loss problems. Moreover, benefiting from the hydrogen bonds between PVA chains, PAAm chains, and MXene nanosheets, the composites have self-healing capability, i.e., the conductivity quickly (within 3.1 s) recovers to the original standard after cutoff. The high conductivity of MXene makes the prepared sensor highly sensitive to strain, so the wearable system can be used as a strain sensor to monitor human motion. Additionally, the hydrogel composite exhibits a low-temperature-tolerant behaviour which is anti-freezing even at extremely low temperatures (−40 °C) (Figure 4o) [180,181], and this strong anti-freezing capability allows this wearable system to be used in extreme environments, expanding its potential applications [182].

### 2.3. Other Parts in Wearable Systems

Advanced wearable systems for health monitoring are generally equipped with wireless signal transmission systems, energy supply systems, and terminal signal analysis systems, and these systems enrich the function of wearable systems [3,24,29,31]. Fully integrated wearable systems have gradually been commercialized. In addition, advanced wearable health-monitoring systems have a profound influence on developing personalized medical and treatment research.

#### 2.3.1. Data Communication System

**Figure 5:**
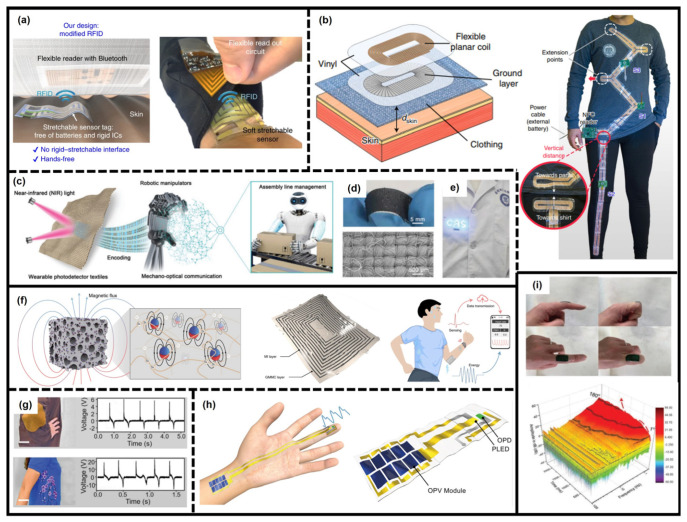
The novel data communication system, energy supply system, and data analysis system in wearable systems. (**a**) Schematic illustration and photograph of a bodyNET sensor system. Reproduced with permission from ref. [40]. Copyright 2019 Nature Publishing Group. (**b**) Schematic illustration of a wearable NFC resonator unit (**left**) and photograph of NFC network integrated on shirt and pants. Reproduced with permission from ref. [42]. Copyright 2021 Nature Publishing Group. (**c**) Schematic illustration of a light–machine interface platform. (**d**) Optical image of wearable photodetector (**top**) and SEM image of Te@TeSe assembled with carbon fibre fabric textile and Ag nanowires (**bottom**). (**e**) Optical image of wearable photodetector textile on clothes. Reproduced with permission from ref. [183]. Copyright 2021 Wiley-VCH. (**f**) Schematic illustration of a magnetoelastic system and its application in health monitoring. Reproduced with permission from ref. [184]. Copyright 2021 Nature Publishing Group. (**g**) A wearable TENG biomechanical energy-harvesting system. Reproduced with permission from ref. [185]. Copyright 2019 Wiley-VCH. (**h**) Schematic diagram of a self-powered photoplethysmograpm sensor on human hands. Reproduced with permission from ref. [186]. Copyright 2021 Nature Publishing Group. (**i**) Three-dimensional surface plot for continuous angular movement of finger. Reproduced with permission from ref. [187]. Copyright 2021 Wiley-VCH.

Communicating data wirelessly is a key technology in the broader use of wearable systems, and conventional wireless data communication systems such as NFC, RFID, antenna, and analogue-to-digital converters (ADCs) have matured in rigid electronic devices [38]. However, applying these data communication systems to wearable systems requires additional considerations, such as the flexibility and robustness decay problem, because of the rigid integrated circuit (IC) chips used in these systems. Niu et al. reported a bodyNET system which was separated into two parts, i.e., the flexible reader with Bluetooth on clothes and a stretchable sensor tag on the skin (Figure 5a) [40]. The data collected by stretchable sensor tags could be transmitted via RFID to flexible readers, and then via Bluetooth to terminals. All the rigid components (i.e., IC chips and batteries) were removed to prevent rigid–stretchable interfaces caused by direct contact with skin, the stress concentration was restrained and the robustness of the wearable system was improved. This bodyNET system based on RFID communication can be used to monitor and analyse pulse, breath, and body motion simultaneously and continuously.

Recently, Hajiaghajani et al. proposed that the RFID and Bluetooth approaches need a high power supply and the data security level is low [42]. Thus, to address this issue, the authors limited the operational range of communication links (i.e., NFC), and a network using a surface plasmon-like metamaterial was made and attached to clothes (Figure 5b). Then, a textile-integrated NFC multibody area network was reported, which can realize long-distance communication between multiple objects through overlaying NFC arrays which are discrete, anisotropic, and magneto-inductive. Due to the advantages of modularity, this network is tuneable and expands according to the user’s needs, and the energy consumed, which is low, can be supplied using NFC, liberating this NFC network from the constraint of batteries [188,189]. The temperature and human motion are accurately monitored and accorded by this NFC wearable system, providing a new method for advanced wearable system design.

Different from traditional communication methods, Li et al. proposed an optical communication system using near-infrared (NIR) light as a medium of transmission [183]. A self-powered wearable system can be built based on the Te@TeSe photodetector textile, and the wearable system can manipulate robotic arms’ actions, following instructions which are commanded by NIR light. Although this wearable system realizes mechano-optical communication, the data transmission is non-reversing, but the data feedback process is a still challenge for using light as a wireless communication way.

#### 2.3.2. Energy Supply Systems

The energy supply of conventional rigid electronic devices/systems generally depends on batteries or an external alternating current (AC) power source, but in wearable systems, the rigid batteries and circumscribed electric wire impair the flexibility/stretchability and portability to a great extent. Moreover, the charging or replacement process and safety problems make traditional rigid batteries unsuitable in wearable systems. Therefore, flexible, sustainable, and portable energy supply systems are desirable for wearable systems [190,191]. The above-mentioned data communication systems simultaneously have energy supply functions, and besides these examples, the wearable systems can also harvest mechanical, light, or thermal energy from the ambient environment. Solar cells, TENG, piezoelectric nanogenerators (PENG), and supercapacitors are common flexible energy supply systems in wearable systems at present, and self-powered wearable systems can be applied to long-term health monitoring [43].

As shown in Figure 5f, Zhou et al. designed a stretchable magnetoelastic generator and used this generator as a wearable system’s power generator and biomedical sensor [184]. The micromagnets are dispersed in a silicon matrix and a giant magnetoelastic effect inside a stretchable system is established with a high magneto–mechanical coupling factor. According to the wavy chain model built by authors, mechanical stress can change the spacing of the micromagnet and dipole alignment, which induces magnetic field altering, and this altering enables biomechanical-to-electrical energy conversion [192]. Wearable systems always work in a complex environment that contains strain, deformation, and/or press, thus this mechanical-induced generator can provide a stable energy supply. The short-circuit current intensity can reach 3.27 mA/cm^2^ and the 20.17 W/m^2^ power can be collected from body movement. Furthermore, this magneto–mechanical generator is used as a self-powered, water-resistant, wearable wrist pulse-monitoring system, and the swimmer’s wrist pulse is accurately recorded.

TENG and PENG are also two kinds of generators converting mechanical energy into electric energy. TENG energy supply systems have several advantages, such as low cost, great reliability, high efficiency, etc., and these merits let TENG provide high and steady output power to systems [193]. Due to the wide use of elastic polymers in TENG, the flexible and stretchable TENG is distinctly better than rigid batteries. Therefore, the research focusing on TENG has been epidemic currently, and multifarious wearable systems based on TENG are devoted to human health monitoring [194]. Medeiros et al. described a self-powered wearable system based on TENG [185]. The energy is harvested from human motion and the power density can reach 600 μW/cm^−2^. The wearable system was fabricated on cloth as exhibited in Figure 5g, and the self-powered, breathable, waterproof system is also highly sensitive to touch.

Solar energy is an abundant green power, and wearable systems can be actuated by solar energy through photovoltaic devices. Jinno and coworkers reported an ultra-flexible photonic skin powered by organic photovoltaic, realizing biosignal detection and monitoring (Figure 5h) [186]. The fully flexible organic photovoltaic modules are connected to flexible organic light-emitting diodes (OLED) and photodiodes (OPD). The integrated optical sensing system performs impressive sensing performance, and the blood pulse signals are detected on human hands.

#### 2.3.3. Data Analysis Systems

With the rise in artificial intelligence, the sensing systems no longer just sense but are able to “feel” the stimulus. The machine learning process trains the sensor with a mass of sensing data, and then the sensor systems can distinguish and recognize the type of stimulation [195]. The advanced wearable system, which is equipped with high-performance sensor systems and data analysis systems, exhibit accurate recognition functions. For example, Horev et al. have used a short-term fast Fourier transformation method to extract the characteristic value from original sensing signals (Figure 5i), and the frequency at maximum amplitude is viewed as a classification basis using principal component analysis (PCA) as a classification method [187]. The 3D spectrogram surface plot is shown for continuous angular movements (1–180°), which can be used in real life to precisely determine the different complex body movements.

There is a typical characteristic for sensing signals: the obtained signals always follow time series, whatever the type of signals. The methods to process and analyse sensing data that follow time series can be mainly classified into two categories, named step-by-step and end-to-end [196,197]. The above mentioned example is a responsive step-by-step method, and using a step-by-step method to analyse the sensing signals usually relies on feature vectors/values. Via a specific algorithm, the feature vectors are extracted from original sensing signals to form hypervectors, which can be used as a classification standard for comparing with training sample databases by the algorithm (such as PCA and discriminate factor analysis (DFA)), and then the state of the sample is recognized. The advantages of the step-by-step method are apparent, e.g., the algorithm target of each step is clear and a small number of hypervectors in each step effectively decreases the calculated amount; the extracted feature vectors possess actual meaning (such as the frequency in the aforementioned example), facilitating analysis process; fewer training samples are needed using this method. However, the parameter of the algorithm needs to be adjusted in each step, causing tedious work, and the feature vectors should be designed by specialists due to their actual meaning, hindering their development to some extent.

The end-to-end analysis method is a deep learning method in which a Convolutional Neural Network (CNN) is used to directly process sensing signals, and the results are contrasted with a training sample database to identify the signal state [198]. Compared with step-by-step, the end-to-end method, with no requirement for feature vectors, is a more automatic method to analyse data like a “black box”. Even though the end-to-end method can automatically learn a relatively ideal feature network structure, many samples are needed to ensure the correctness of the CNN. Moreover, the construction of CNN also requires the assistance of a specialist, and due to the learned feature factor being hard to explain, the analysis and adjustment process of an end-to-end method is nonrepresentational compared with a step-by-step method.

Thus, how to select the data analysis method determines the recognition quality of wearable systems, and ensures the correctness of results; the effective, sufficient, and real training samples are the most important principle to observe. A satisfactory data analysis system can endow wearable systems with “wisdom”, and smart wearable systems will become the main force in health monitoring in the next generation.

## 3. Wearable Health-Monitoring Systems

### 3.1. Anatomical Movement Monitoring

Developing high-performance wearable movement-monitoring systems plays an essential role in action recognition, motor function assessment, and dexterous human–machine interaction, which is significant for rehabilitation and intelligent prosthetics [10,199,200]. A sensor for monitoring anatomical movements of the human body must be sensitive to the applied force in three major planes (i.e., sagittal, coronal, and axial) and three major axes (i.e., sagittal, coronal, and vertical). The anatomical movements can be classified into two categories, i.e., slight movements (e.g., muscle movements with no joint rotation) and full range movements (e.g., joint rotation). The traditional movement-monitoring methods rely on machine vision (or optical) systems or angle encoders, but these methods suffer from the limitation of freedom leading to discomfort, even joint injury in long-term application [201,202]. The flexible/wearable sensors/systems (i.e., force-based sensors, soft strain sensors, micro inertial sensors, and surface electromyography sensors) may overcome this problem, and these sensors have been commonly used due to their ability to gain direct measurement of body segment movement [49].

**Figure 6:**
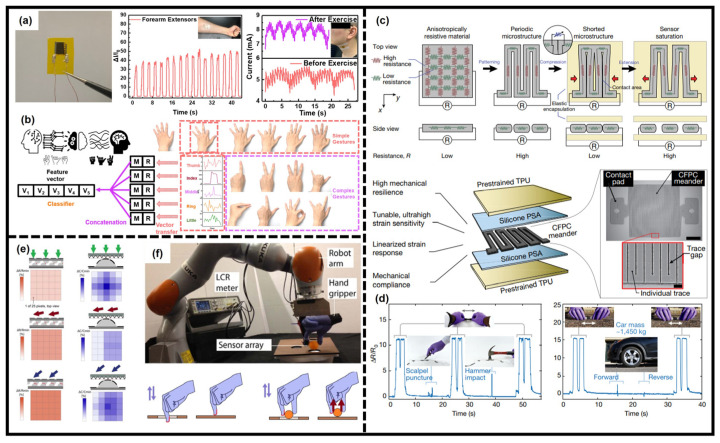
The wearable systems for human movement monitoring. (**a**) Photograph of a dual-microstructure MXene-based piezoresistive pressure sensor and its performance in muscle movement, respiration, and carotid artery monitoring. (**b**) Gesture recognition system based on this wearable sensor. Reproduced with permission from ref. [62]. Copyright 2021 The Royal Society of Chemistry. (**c**) The operation principle of a strain-mediated contact in anisotropically resistive structures (SCARS) sensor (**top**) and schematic illustration of the sensor. (**d**) Demonstration of sensor resilience to punctures (**left**) and high loads (**right**). Reproduced with permission from ref. [205]. Copyright 2020 Nature Publishing Group. (**e**) Schematics showing the comparation of two different structures in detecting the direction of applied pressure. (**f**) Experiments with sensor mounted on a robot arm. Reproduced with permission from ref. [87]. Copyright 2021 American Association for the Advancement of Science.

To monitor all kinds of anatomical movements, the sensitivity and detection range is equally important. However, usually, only one property (sensitivity or detection range) is improved while another is ignored, rather than being achieved simultaneously. Guo et al. proposed that the sensitivity and detection range of piezoresistive monitoring devices are strongly related to the microstructures, and the pressure sensor with surface microstructure may have high sensitivity and the internal microstructure can improve the detection range [62]. Thus, the authors designed a dual-microstructure pressure sensor for synchronous anatomical movement monitoring. The surface and internal microstructures were constructed by removing the sacrificial template, and the final pressure sensor can simultaneously achieve high sensitivity (401.01 kPa^−1^, 0–12 kPa) and wide detection range (1.96 Pa to 100 kPa) with real-time performance (responses within 103 ms) and great stability over 6000 cycles (Figure 6a). The wearable device based on this high-performance pressure sensor enables the detection of a wide range of movements (e.g., flexion and extension of the elbow, wrist, and fingers) and highly sensitive detection (e.g., movements of masticatory muscle, deltoid, and forearm extensor). With the assistance of data mining methods, such as machine learning, the gesture behavioural information is also extracted by a wearable gesture recognition system (Figure 6b) [196,197]. The multiple physiological signals (e.g., movements, respiration, and carotid artery) can be synchronously monitored and decoupled using a Fourier transform filter.

Strain sensors are always used to detect mechanical strain and can be also applied to monitor human movements. However, when using the strain sensor to monitor anatomical movements, the off-axis deformations, such as bending, torsion, and pressure deformation, are disruptive to strain sensing [203,204]. Meanwhile, there are many adverse conditions during health monitoring, e.g., impacts, overextension, and punctures, which are also undesirable; thus, the ability of wearable strain sensors to decouple or reject these off-axis deformations is advantageous. Recently, Araromi et al. presented a highly sensitive strain detection mechanism, based on strain-mediated contact in anisotropically resistive structures (SCARS) (Figure 6c) [205]. The sensing layer is made of anisotropic-resistive material and patterned into periodic microstructure, which can be compressed or extended under mechanical strain. The high sensitivity (GF > 85000) was achieved, and using high-strength conductors, the strain sensing system was resilient to adverse off-axis loading. The low resistance pathway contacts/separates with adjacency under compression/extension, and the resistance of the sensing layer changes and the Ohmic resistance shows a linear change under applied strain with negligible bending or twisting deformation (Figure 6d). The discrete gestures and continuous hand motions can be predictively tracked and classified via the detection of small muscle movements in the arm using this wearable strain sensing system.

The ability of wearable systems to measure and discriminate normal and shear forces is essential to provide texture and slip information [85,206,207]. Normal and shear forces and the direction of pressure are needed for health monitoring or auxiliary machinery, which are feedback data required for many activities such as holding goods or inserting a key in a lock. However, the shear force direction of applied pressure is difficult to obtain and recognize. Inspired by biological skin, Boutry et al. reported a soft system composed of an array of capacitors, which can measure and discriminate normal and tangential forces in real-time [87]. The capacitors array was designed into a specific microstructure (i.e., the top layer is a pyramid shape and the bottom layer is a hemispherical shape) (Figure 6e), and the micro-structured sensor array is sensitive to normal pressure, shear force, the composite force, and bending. As shown in Figure 6f, a robot arm holding a hand gripper equipped with this device can imitate human tactile sense to shear force feedback.

### 3.2. Arterial Pulse Pressure Monitoring

Arterial pulsation is caused by the ejection of blood from the left ventricle into the aorta, travelling to arteries throughout the whole body [48]. The blood flows back to the heart with the aorta contracting when the ventricles diastole, and when the ventricles systole, the blood is sent to the rest of the body, caused by the relaxation of the aorta. The arterial pulse can be detected by a form of pressure fluctuation contributed by the blood movement. During the diastole and systole, the change in blood flow, caused by the concentration and dilation of the blood vessel, can influence the blood volume, and then change the intravascular pressure pulsation [208,209]. The blood is pumped throughout the body by vasoconstriction, and the arterial vessels are straightened and narrowed, increasing arterial pulse pressure, which is inverse when the arterial vessels dilate. Blood is the most important body fluid, which carries essential substances and passes through all organs, supplying oxygen and nutrients. Meanwhile, the blood flowing through the organs can carry out waste and regulate temperature, and the blood circulatory system is connected to most of the organs, which can reflect the healthy condition of other organs [210,211,212]. For example, cardiovascular diseases, as serious and fatal diseases, can be monitored and prevented through proactively and continuously monitoring blood pressure (BP) [213]. Nowadays, cuff sphygmomanometers are the most commonly used method to measure BP, but due to the discomfort and bulkiness, this method is unable to continuously monitor in daily life [214,215].

**Figure 7:**
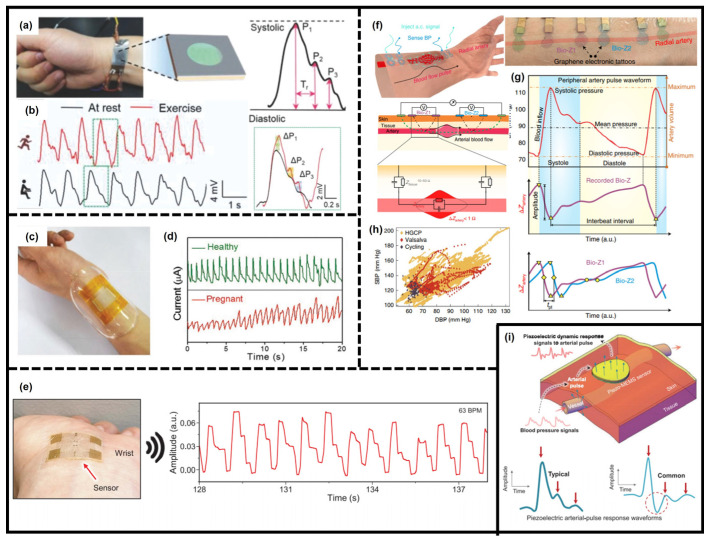
The wearable systems applied in arterial pulse pressure monitoring. (**a**) Photograph of a patchable sensor attached on a wrist to measure the radial artery (**left**) and three typical types in radial artery measurements. (**b**) Two characteristic signals before and after exercise and magnified curve of pulse wave signal of heart rate and type of blood pressure. Reproduced with permission from ref. [216]. Copyright 2018 Wiley-VCH. (**c**) Photograph of a device for detection of wrist pulses and (**d**) original wrist pulses signals of a non-pregnant person and a pregnant woman. Reproduced with permission from ref. [63]. Copyright 2014 Wiley-VCH. (**e**) A wireless pulse measurement using GaN SAW wearable system. Reproduced with permission from ref. [38]. Copyright 2021 American Association for the Advancement of Science. (**f**) Illustration of bioimpedance blood pressure measurement. (**g**) Correlation between arterial blood pressure and bioimpedance. (**h**) Recognition performance of bioimpedance-monitoring system. Reproduced with permission from ref. [218]. Copyright 2022 Nature Publishing Group. (**i**) Illustration of piezoelectric dynamic response to arterial pulse and typical waveform. Reproduced with permission from ref. [219]. Copyright 2022 Wiley-VCHv.

As a form of physiological movement, the essence of arterial pulse monitoring is sensing pressure change, so the high-performance pressure sensor mentioned above can also be used to monitor human BP. Chun et al. reported a self-powered mechanoreceptor sensor which can accurately record the radial artery pulse (Figure 7a) [216]. The collected sensing signals show a typical shape which reveals three pulse waveforms [i.e., percussion wave (P-wave), tidal wave (T-wave), and diastolic wave (D-wave)], and physiological information can be obtained from these signals, such as radial artery augmentation index (AI_r_), radial diastolic augmentation index (DAI), pulse interval, round-trip time (a reflected wave from hand periphery) (T_r_), etc., among which the AI_r_, DAI, and T_r_ are common parameters to diagnose arterial stiffness [217]. Moreover, the waveform of signals is different at rest and after exercise, meaning the state of the artery pulse is changed due to the different heart rates (Figure 7b).

The physiological information conveyed by pulse can be used as a reference to diagnose cardiovascular disease. For instance, atherosclerosis leads to pathologic change in arterial pulse and affects BP even in the very early stage, which is asymptomatic in other symptoms. Wang et al. presented a microstructured PDMS/CNT pressure sensor with excellent sensitivity, and this sensing device provides a noninvasive way to diagnose cardiovascular diseases (Figure 7c,d) [63]. The pulse signals from non-pregnant people and a pregnant woman were measured, and through extracting feature information in pulse signals (e.g., pulse frequency, P-, T-, and D-wave), the pregnant woman could be accurately distinguished from those not pregnant.

In daily life monitoring, the comfort and robustness of pulse sensing systems are indispensable, and the wearable system should fit the body as closely as possible, which may cause skin inflammation. Thus, breathability is always needed for long-term monitoring [48]. The aforementioned GaN surface acoustic wave wearable system possesses high sensitivity and is comfortable to wear (Figure 7e), and this wearable system can also record human pulse [38]. Remarkably, the system can be worn for 17 h/day and the monitoring can maintain over 7 days, which demonstrates the reusability and long-term service capacity of this wearable system. This outstanding wearable system offers a versatile biomedical sensing platform for health monitoring and disease diagnosis.

The existing cuffless BP-monitoring methods can also rely on optical or bioimpedance besides pressure [220,221]. A commonly used optical method is photoplethysmography (PPG), which consists of a light emitter and detector. The light with a certain wavelength is emitted from the emitter and directed towards the skin surface, and then the detector monitors transmission light and reflected light. Due to the damping intensity caused by skin, bones, veins, muscles, and other tissues being essentially constant, the absorption of detected light is only influenced by the change in blood volume and arterial systole and diastole, in other words [48]. However, the sensing depth of optical-monitoring methods are limited to capillary regions by shallow penetration of light. Moreover, the tonometry methods relying on pressure sensors require a bone presence which localizes the placement area of wearable systems. Kireev and coworkers introduced a wearable bioimpedance BP-monitoring system that is made of graphene electronic tattoos (Figure 7f) [218]. The bioimpedance-monitoring method has the capability to sense buried arteries’ pulse using electrical currents with deep penetration. The change in pressure during arteries’ systolic and diastolic phases is revealed by the amplitude change in bioimpedance. Briefly, the blood vessels, containing rich ionic solution, possess better conductivity than surrounding fat and muscle cells, and the arterial volume is correlated with BP, which determines the impedance. The characteristic features (e.g., systolic pressure, diastolic pressure, interbeat interval, and pulse transit time) are precisely monitored and recorded (Figure 7g). Furthermore, the machine learning model was created with these characteristic features, enabling this wearable system successfully to monitor the BP of volunteers under a different state of motion (Figure 7h).

It is worth noting that there is an exceptional case where, when using piezoelectric devices to monitor arterial pulse, the accuracy of BP evaluation is limited. Because of the distance error of located sensors and time synchronization error, the waveforms of signals, obtained by piezoelectric devices, are disturbed and anamorphic compared with true BP signals (Figure 7i) [222,223,224]. Thus, the use of piezoelectric methods for BP monitoring is controversial. In order to eliminate this problem, the reason causing distortion should be found and resolved. Yi et al. elucidated the relationship between BP waveforms and the thickness of the piezoelectric functional layer and eliminated the signal distortion [219]. Via integration, transition correction, and direct correlation, the arterial pulse signals can be truly monitored and recorded using piezoelectric wearable devices. This approach eliminates the controversy over using piezoelectric devices to monitor arterial pulse and can potentially be used to achieve daily health monitoring.

### 3.3. Electrophysiological Signals Monitoring

Electrophysiological signals, as a vital information medium, are exchanged between the nerve systems and various motor and sensory end-plates. Most of our living activities are controlled by the nervous system through electrophysiological signals, such as advanced cognitive activities (e.g., thinking and memory) and daily movements (e.g., muscle movements), and meanwhile, the sensory information is transferred back to the nervous systems [30]. Hence, through monitoring electrophysiological signals, the neurological status of patients can be observed in real-time, which is needful for many diseases such as Parkinson’s disease, epilepsy, heart failure, and hypertension. Electroencephalography (EEG), electrocorticography (ECG), and electromyography (EMG) are the most representative electrophysiological signals noticed by people [10].

**Figure 8:**
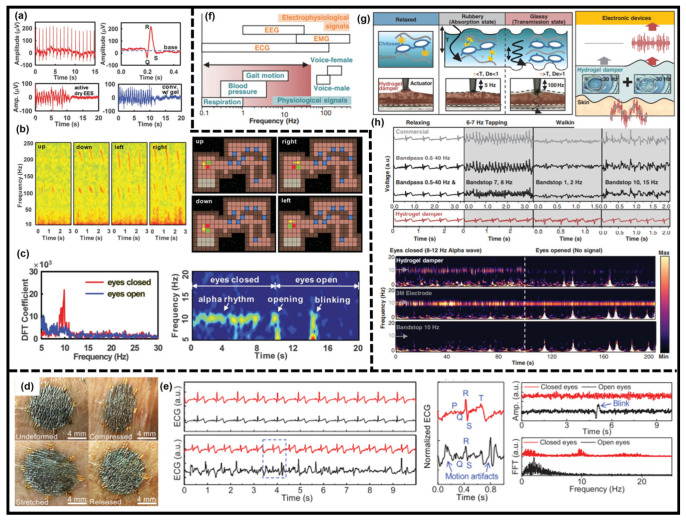
The wearable systems used in electrophysiological signals monitoring. (**a**) ECG (**top**) signals measured with a wearable EES and obtained EMG (**bottom**) signals compared with conventional sensors. (**b**) Spectrogram of the EMG data mounted on the neck during vocalization of four different words (**left**) and simulated video game control by EMG data. (**c**) EEG alpha rhythms measured by EES. Reproduced with permission from ref. [23]. Copyright 2011 American Association for the Advancement of Science. (**d**) Skin−gate van der Waals transistor on human skin under different deformation. (**e**) The ECG signals measured by the skin−gate transistor (rad line) and Ag/AgCl electrode (black line) (**left**) and recorded EEG signals (closed eyes and open eyes) (**right**). Reproduced with permission from ref. [227]. Copyright 2022 American Association for the Advancement of Science. (**f**) Representative frequency ranges of human mechanical and electrophysiological biosignals. (**g**) Schematic illustration of selective biophysiological signal detection through the hydrogel damper and the selective damping mechanism. (**h**) The ECG and EEG signals measured with the hydrogel damper. Reproduced with permission from ref. [228]. Copyright 2022 American Association for the Advancement of Science.

At present, the clinical methods to monitor electrophysiological signals rely on traditionally rigid metal electrodes and gel electrolytes, which are typically attached to the skin via tapes, mechanical clamps, or straps. However, there are many issues during long-term application using rigid electrodes and gel electrolytes as monitoring devices, e.g., the unstable electrode–tissue interface, gel electrolyte dehydration, and poor wearing comfort, which may lead to a decrease in signal-to-noise ratio [175,225]. In addition, electrophysiological signal monitoring is always confined to a hospital bed by bulk power supplies and communication components. Thus, advanced wearable systems are exploited to provide long-term stable monitoring methods and ameliorate wearing comfort. The above-mentioned epidermal wearable system (Figure 1e) reported by Kim et al. possesses an electrophysiological monitoring function, and the ECG, EMG, and EEG can be continuously monitored for as many as 6 h [23]. The favourably conformal contact of the wearable system and skin enables this system to maintain satisfactorily stable monitoring performance even when the system is mounted in challenging areas (e.g., elbow). The ECG signals are recorded from the chest, revealing all phases of the heartbeat and including the cardiac wave’s rapid depolarization phenomenon (Figure 8a) [226]. Moreover, the obtained EMG signals are compared with conventional monitoring methods based on gel electrolyte, and the signal quality of the wearable system is remarkably good with commercial electrodes. The EMG signals can be illustrated alternatively, appearing as the spectral content in a colour contour plot, and the speech command is recorded and recognized by a wearable system with EMG signals from the throat, which creates opportunities for human/machine interfaces and controls a computer strategy game (Figure 8b). In addition, the alpha rhythms, as an EEG signal, are only apparent when the eyes close, and the recorded EEG signals show an obvious difference at 10 Hz frequency, which corresponds to alpha rhythm, revealing the excellent performance of this wearable system in electrophysiology monitoring (Figure 8c) [59].

To ensure the conformal contact between wearable systems and skin, Yan et al. reported the design of a wearable system based on van der Waals thin films (Figure 8d) [227]. The stretchability, malleability, and breathability are ensured simultaneously because the staggered nanosheets can freely slide and rotate under deformations. Moreover, the stretchability of thin films allows the necessary deformation of the sensing layer to adapt to the local surface topography. A conformal contact between thin sensing film and skin is shown in Figure 8d, and there is still no separation, even under compressing and stretching. The excellent contact condition guarantees stable interface impedance, and the applied FET can exactly monitor electrophysiological signals. After attaching this wearable FET system to the human skin, the ECG and EEG signals (i.e., alpha rhythms) are monitored and recorded in real-time (Figure 8e). The ECG signals measured by wearable systems show better anti-interference to mechanical motion compared with traditional Ag/AgCl-monitoring devices.

The electrophysiological signals are seriously disturbed by the unexpected movements of patients. As for the human body, mechanical motions are perennial and superimposed on electrophysiological signals [229]. The current methods to avoid disturbing movements rely on signal processes such as bandpass filters, which may result in signal loss [230]. Park et al. were inspired by the viscoelastic cuticular pad in nature (e.g., spider) and presented a wearable electrophysiological signal-monitoring system based on a bandpass filter material [228]. The unexcepted movements are in a low-frequency range, such as walking and respiration are under 30 Hz, but the electrophysiological signals are high-frequency signals (Figure 8f). The bio-inspired material exhibits frequency-dependent phase transition, which leads to the bandpass filter function. In brief, the hydrogel can change from a rubbery to a glassy state when the frequency of applied signals is above 30 Hz and the high-frequency vibration signals (e.g., electrophysiological signals) can be transmitted while filtering low-frequency unexcepted movement signals (Figure 8g) [231]. As a result, the wearable system based on band-pass filter hydrogel serves as a high-quality health monitoring system, and the electrophysiological signals such as ECG and EEG are exactly monitored (Figure 8h).

As long-term, daily, and wearable monitoring equipment, the wearable systems used to monitor electrophysiological signals are always beset by wet conditions, such as sweat or rainwater, which cause signal distortion or damage to devices. Thus, the performance of wearable systems for electrophysiological signal monitoring should be reliable in either dry or wet conditions [232]. Noh et al. have reported a novel copper-meshed carbon black/PDMS electrode which can be used to monitor ECG signals in all water immersion conditions with a superior performance, showing an advisable way to design water-resistant electrodes [233]. Based on this strategy, more eminent electrodes have been designed, and these electrodes ensure the stability of wearable systems used to detect electrophysiological signals, granting them more potential and making them useful in daily monitoring [232,234,235].

### 3.4. Biochemical Monitoring

Wearable biomedical sensing systems have been developed for disease diagnosis and health monitoring, and unlike traditional diagnostic methods (i.e., in vivo/vitro pathological and clinical examinations), the wearable systems are portable, comfortable, and low-cost [101]. Various diseases have been studied and recognized by wearable biochemical-monitoring systems, such as cancer (e.g., lung cancer, colorectal cancer, bladder cancer, etc.), neuropathic disease (e.g., atypical parkinsonism, idiopathic Parkinson’s disease, multiple sclerosis), and chronic disease (e.g., chronic kidney disease, preeclampsia, hypertension, hyperglycaemia) [3,236,237]. The biomarkers are commonly detected as monitoring parameters of wearable biochemical-monitoring systems, and the biomarkers which are specifically associated with diseases can be found in skin odour, breath, and body fluids (e.g., sweat, tears, saliva, blood, etc.) [101,238,239].

**Figure 9:**
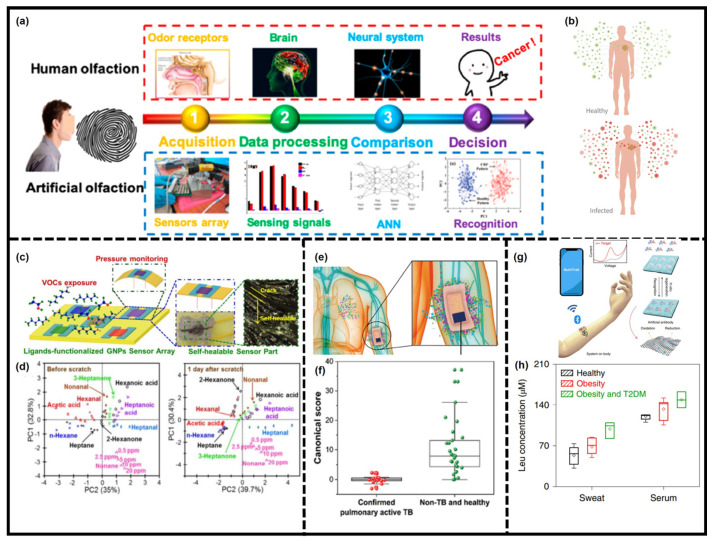
The wearable systems used in biochemical biomarkers monitoring. (**a**) Schematic of breath diagnosis. Reproduced with permission from ref. [240]. Copyright 2019 Wiley−VCH. (**b**) Schematic of the VOC−based disease monitoring with wearable systems. Reproduced with permission from ref. [10]. Copyright 2022 The Royal Society of Chemistry. (**c**) Schematic of a self−healable VOCs sensor array and (**d**) PCA of sensing response from this sensor array to 11 types of VOCs emitted from healthy volunteers’ skin. Reproduced with permission from ref. [241]. Copyright 2016 American Chemical Society. (**e**) Schematic of a VOCs−monitoring wearable device and (**f**) boxplot of the canonical score of DFA model of sensing signals obtained from this wearable device recognizing patients. Reproduced with permission from ref. [242]. Copyright 2021 Wiley−VCH. (**g**) Schematic of a wearable nutrient-monitoring systems and (**h**) boxplot of measured Leu levels in sweat measured by this wearable chemical sensor. Reproduced with permission from ref. [57]. Copyright 2022 Nature Publishing Group.

As shown in Figure 9a,b, the special (bio)chemical molecular biomarkers contained in body fluid can be sensed and back-fed by sensors in wearable systems, and then the data analysis systems diagnose the diseases [10,240]. Moreover, disease diagnosis via biomarkers is difficult because of the small sample volumes, external interference, and dilution of biomarkers, and, thus, the performance of wearable monitoring systems determining the diagnosis results should be continually improved. Recent advances in artificial intelligence are helpful for (bio)chemical physiological signal monitoring using wearable systems. In short, a great deal of (bio)chemical sensing information can be collected from volunteer patients and healthy people to set up a database (as mentioned in Section 2.3.3), and the different health state of people is diagnosed by referring to the sensing information database.

A sensor array, proposed by Jin et al., consists of five kinds of gas sensors based on functionalized gold nanoparticles, and the sensor array can detect 11 kinds of volatile organic compounds (VOCs) (Figure 9c) [241]. In addition, this wearable sensor array is self-healing to enable extended usage periods. As a result, the wearable sensor array possesses a low detection limit, high sensitivity, and excellent stability, which can be maintained after self-healing. The PCA algorithm was used to discriminate the biochemical information contained in the sensing signals, and the different health states of volunteers were diagnosed (Figure 9d). The satisfactory discrimination features of these wearable systems presage a new type of wearable biochemical signal-monitoring system, with great prospects in smart wearable health-monitoring systems.

In another example, the functionalized gold nanoparticles were also used to detect the VOCs in human skin odour, enabling the diagnosis of tuberculosis. The sensors were integrated into a wearable device, and the tuberculosis-specific VOCs were detected by these sensors from the skin’s headspace [242]. The sensing information was collected from 29 healthy volunteers and 18 confirmed active pulmonary tuberculosis patients (Figure 9e,f), and the Discriminate Factor Analysis (DFA) algorithm, the database of which included 475 samples’ information (299 healthy volunteers and 176 confirmed active pulmonary patients), was introduced to evaluate the health state of the target. The results showed that the diagnostic accuracy of this wearable system reached 89.4% with an 86.2% specificity and a 94.4% sensitivity, meaning that tuberculosis can be accurately detected and monitored with this wearable system.

The biochemical information of the human body is not only contained in VOCs but also in body fluid, which can be also monitored by wearable systems. Recently, Wang et al. designed a wearable electrochemical biosensor to monitor metabolites and nutrients (Figure 9g,h) [57]. The authors tactfully presented a biosensor based on graphene electrodes that can be repeatedly regenerated and functionalized with an antibody which is specific to metabolite, distinguishing it from classic single-use biocompatible sensors with molecularly imprinted polymer or antibodies. With the help of a microfluidic sampling way, the sweat was analysed by the wearable system, and the results showed that the amino acid levels in serum and sweat in healthy volunteers and patients with obesity and T2DM were different, which assessed the risk of metabolic syndrome.

## 4. Conclusions and Perspectives

In this review, we summarized some representative achievements and recent progress of wearable systems for health monitoring, and the overview was focused on materials, systems integrating, and (bio)physiological signal monitoring. We also elaborated on the materials selection strategies of each unit in wearable systems, including flexible substrate, sensing materials, and conductive electrodes, and then the other integrated units used to transmit data and energy were also introduced. In addition, the applications of wearable systems in health monitoring, e.g., anatomical movement, arterial pulse, and electrophysiological and (bio)chemical physiological signals, were described in detail. However, there are still some remaining challenges in developing wearable systems, and we outline several issues with our perspectives as follows.

(1) Materials. Recent advances in nanomaterials facilitate the development of wearable systems. As mentioned above, different characteristics are needed for materials used in different parts of wearable systems, so the technological trends of materials in wearable systems are various, including flexibility/stretchability, conductivity, sensitivity, etc. Using composite materials is a feasible way to multidimensionally improve the performance of wearable systems, but the compatibility issue between different materials is an obstacle, so the optimal combination mode is critical to exploit advanced composite materials which can be used in wearable systems. Even though regulating the percolation threshold of materials and functionalizing are two effective ways to improve compatibility, a sound strategy is still highly required to solve this issue.

(2) Systems integrating. Wearable systems have been endowed with more and more functions, and these advanced technologies, such as self-power/solar cell systems, wireless interaction, and artificial intelligence, elevate the portability, practicability, and intelligence of wearable systems. However, signal interference is a common problem when many function parts are integrated into a wearable system, which may influence the authenticity of signals obtained by wearable systems. Moreover, the position of the human body or clothes of different units in wearable systems should be prudently arranged, and a reasonable layout can minimize the influence caused by mechanical deformation while maintaining the sensing performance. Last but not least, novel systems with unique functions are focused on now, such as sensing signal amplification or pretreatment systems and automatic alarms, and, thus, more special systems are needed towards various applications. In addition, during the machine learning process, it should avoid excessive artificial correction in data analysis.

(3) Health monitoring. Conformability, safety, and stability are the most essential properties of wearable systems for health monitoring, so the inter-contact between wearable systems and human skin must be safe, nontoxic, and comfortable. Moreover, artificial intelligence is a terrific auxiliary means for wearable systems, which has been widely used to diagnose the health state of people by analysing the sensing data. Due to the result of the algorithm being directly concerned with sample capacity, the database must contain effective, sufficient, and real training samples to ensure the reliability of the learned model, so collecting sample data is a long way from setting up a satisfactory database. In addition, excessive artificial data correction should be avoided when artificial intelligence is used to analyse data.

In conclusion, smart wearable systems for health monitoring are meaningful in disease diagnosis, monitoring, and treatment. Using our advanced materials and high-tech methods to develop wearable systems may open the next generation’s lifestyle, and good health monitoring can effectively improve the quality of life of the human race.
